# Therapeutic Evaluation of Rosmarinic Acid in a Rat Model Combining Hypertension, Diabetes, and Nephrolithiasis

**DOI:** 10.3390/ph18121773

**Published:** 2025-11-21

**Authors:** Anelise Felício Macarini, Mariana Zanovello, Anelize Dada, Rita de Cássia Vilhena da Silva, Rogério Corrêa, Priscila de Souza

**Affiliations:** Programa de Pós-Graduação em Ciências Farmacêuticas, Universidade do Vale do Itajaí, Itajaí 88302-901, Brazil

**Keywords:** polyphenol, blood pressure, hyperglycemia, oxalate crystals, comorbidity model

## Abstract

**Background**: Cardiometabolic disorders such as hypertension and diabetes are major contributors to chronic kidney disease and often coexist, amplifying dysfunction and metabolic imbalance that favor renal injury and nephrolithiasis. Although pharmacological therapies exist for blood pressure and glycemic control, few target these mechanisms simultaneously. Rosmarinic acid (RA), a polyphenolic compound, exhibits antioxidant, anti-inflammatory, and nephroprotective effects, but its role in combined models of hypertension, diabetes, and nephrolithiasis remains unexplored. **Objectives**: This study investigated the therapeutic potential of RA in an experimental model combining hypertension, diabetes mellitus, and nephrolithiasis. **Methods**: Male Wistar spontaneously hypertensive (SHR) and normotensive rats were assigned to eight groups, including controls, comorbid groups, and treatments with RA (10 mg/kg) or hydrochlorothiazide (HCTZ; 5 mg/kg). Diabetes was induced by streptozotocin and nephrolithiasis by ethylene glycol plus ammonium chloride. Hemodynamic, biochemical, oxidative stress, and histological parameters were assessed. **Results**: SHR exhibited sustained hypertension, further aggravated by diabetes and nephrolithiasis. RA stabilized arterial pressure progression, whereas HCTZ significantly reduced blood pressure. RA and HCTZ treatments decreased urinary calcium oxalate crystal formation by 47.34 and 58.99%, respectively, and partially restored renal morphology. RA restored superoxide dismutase activity, preserved nitrite levels, and reduced lipid peroxidation, indicating antioxidant and endothelial protection. Neither treatment normalized glycemia or fully recovered renal function. Histological analysis showed attenuation of tubular and glomerular alterations in treated groups, particularly with RA. **Conclusions**: Overall, RA exerted antioxidant, nephroprotective, and antilithogenic effects in this complex comorbidity model, supporting its potential as a complementary therapeutic agent in chronic metabolic and renal disorders.

## 1. Introduction

Non-communicable chronic diseases (NCDs) are a major public health challenge worldwide, significantly affecting patients’ quality of life and burdening healthcare systems [1]. Among these, diabetes mellitus (DM) and hypertension stand out due to their high prevalence and frequent coexistence, often creating a complex clinical scenario [2]. These conditions are also associated with an increased risk of kidney stone formation, which may result from pre-existing metabolic disturbances or poor dietary habits [3,4].

DM is characterized by persistent hyperglycemia caused by defects in insulin secretion and/or action, and is associated with microvascular complications (retinopathy, nephropathy, neuropathy) and macrovascular complications (stroke, coronary artery disease) [5]. HTN, in turn, is defined by sustained elevation of systemic blood pressure and is a major risk factor for cardiovascular and renal diseases, as it can lead to hypertensive nephropathy [6,7].

Nephrolithiasis, or kidney stone disease, results from the formation of solid aggregates composed of minerals and salts that accumulate in the kidneys. These stones can cause intense pain, urinary tract obstruction, and infections. Both DM and hypertension are established risk factors for kidney stone formation, while the presence of kidney stones can also indicate underlying metabolic disorders [3,4]. Proper management of these conditions involves lifestyle changes, including a balanced diet, adequate hydration, and physical activity. However, patient adherence is often low, and diets high in sodium, refined sugars, and saturated fats remain common, contributing to the development or worsening of DM, hypertension, and nephrolithiasis [6,7,8,9]. Pharmacological treatments are condition-specific and may have adverse effects, for instance, hydrochlorothiazide, a widely used antihypertensive drug, has been associated with increased risk of developing DM [10].

Given these challenges, there is growing interest in complementary and innovative therapeutic approaches that improve clinical outcomes and patients’ quality of life. Ongoing research is essential for identifying new compounds and optimizing existing therapies, especially those informed by traditional knowledge and the medicinal use of plants. A common compound found in these plants, such as *Rosmarinus officinalis* (rosemary) and *Mentha spicata* (spearmint) [11,12], is rosmarinic acid, which has gained attention for its potential therapeutic effects, mainly anti-inflammatory, but also hypolipidemic, antioxidant, anti-atherosclerotic, anticancer and hepato-protective activities [11]. Furthermore, recent studies have reported the diuretic activity [13] and prevention of calcium oxalate (CaOx) kidney stones, which are the most common clinical type of kidney stones, by rosmarinic acid, both in vitro [14] and in vivo [15].

To investigate the therapeutic potential of RA in a setting that mimics human pathophysiological complexity, an appropriate experimental model is essential. Animal models exploring isolated conditions such as hypertension, diabetes mellitus, or nephrolithiasis are well established in the literature [16,17,18,19]. Although comorbidity models combining hypertension and diabetes, particularly using spontaneously hypertensive rats (SHR) and streptozotocin-induced diabetes, have been previously described [20], models incorporating a third component such as nephrolithiasis remain scarce. This model can provide a more clinically relevant framework for simulating the complex pathophysiological conditions observed in patients with concurrent metabolic and cardiovascular disorders, particularly within high-risk populations.

Therefore, this study aimed to evaluate the effects of rosmarinic acid and hydrochlorothiazide on hemodynamic, glycemic, biochemical, histological, and oxidative stress parameters in hypertensive rats with experimentally induced diabetes and nephrolithiasis.

## 2. Results

### 2.1. Arterial Pressure Results

The analysis of arterial pressure data revealed significant variations among the experimental groups over time, highlighting the impact of comorbidities and the tested treatments.

**Figure 1:**
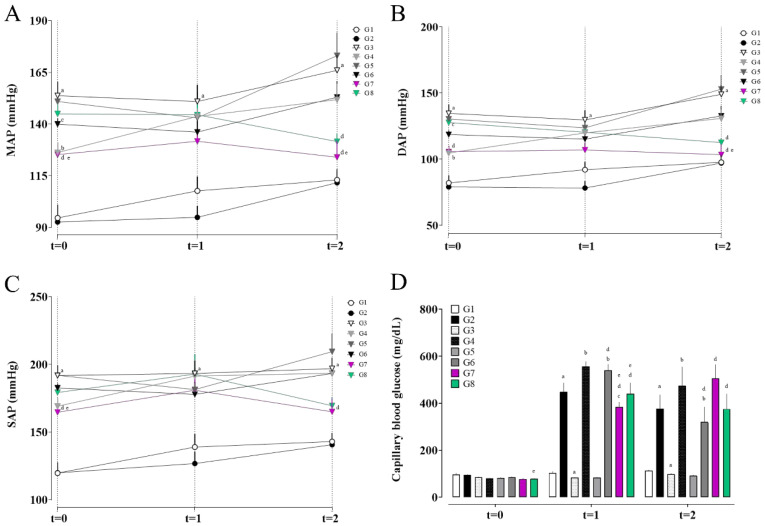
Mean arterial pressure (MAP; **A**), diastolic arterial pressure (DAP; **B**), systolic arterial pressure (SAP; **C**), and fasting blood glucose levels (**D**) in the different experimental groups. (a) G1 vs. G2 and G3; (b) G3 vs. G4, G5, and G6; (c) G4 vs. G6, G7, and G8; (d) G5 vs. G6, G7, and G8; and (e) G6 vs. G7 and G8. Data are expressed as mean ± SEM. Statistical significance was determined by one-way ANOVA followed by Dunnett’s post hoc test. *p* < 0.05 was considered significant.

At baseline (t = 0), the Wistar groups (G1 and G2) exhibited similar mean (MAP), diastolic (DAP) and systolic arterial pressure values (SAP) (Figure 1). In contrast, group G3, composed of spontaneously hypertensive rats (SHR), showed significantly higher MAP, DAP and SAP compared to the Wistar G1 group, as expected [16,21,22]. Groups G4 and G6 displayed lower MAP and DAP than G3 at this timepoint, though still elevated, which may be attributed to individual biological variability.

After seven days of STZ administration (t = 1), the Wistar groups (G1 and G2) maintained similar pressure levels, indicating that diabetes alone did not induce significant hemodynamic changes in these animals. The SHR group (G3) continued to exhibit elevated MAP, DAP and SAP compared to G1.

At the final timepoint (t = 2), following seven days of EG + AC administration and treatment with rosmarinic acid (RA) or hydrochlorothiazide (HCTZ), distinct effects were observed. G5 (SHR with untreated nephrolithiasis) showed the highest values of MAP, DAP and SAP, presenting a 23.56% increase in MAP at t = 2 compared to t = 1, the largest increase among all groups, even though it did not differ statistically from group G3.

In contrast, the groups treated with RA (G7) and HCTZ (G8) showed improvements in pressure parameters. Group G7 exhibited stabilization of mean and diastolic arterial pressure compared to G5 and G6, preventing the progression of hypertension, with a 5.86% reduction in MAP at t = 2 relative to t = 1. Group G8, treated with HCTZ, showed significant reductions in mean and diastolic pressure compared to G5, with the greatest reduction in MAP, with an 8.96% decrease at t = 2 relative to t = 1.

The results indicate that RA (G7) exerts a hemodynamic stabilizing effect, limiting the progression of hypertension without necessarily reversing it, whereas HCTZ (G8) was more effective in the absolute reduction in mean (MAP), diastolic (DAP), and systolic (SAP) arterial pressure. Both treatment groups achieved the highest percentage reduction in systolic pressure, with 8.82% in G7 and 12.09% in G8, followed by reductions in mean arterial pressure, and finally diastolic pressure, with decreases of 5.86% and 3.29% in G7 and 8.96% and 6.48% in G8, respectively.

### 2.2. Fasting Glucose Results

Regarding fasting blood glucose (Figure 1D), at timepoint t = 1 (seven days after streptozotocin administration), pronounced hyperglycemia was observed in the groups that received STZ, confirming the effectiveness of the diabetes induction model.

Group G2 (Wistar + STZ) showed an average capillary glucose level of 448.22 mg/dL, representing a 375.7% increase compared to its baseline value. Group G4 (SHR + STZ) exhibited an even higher value of 555.67 mg/dL, corresponding to a 594.6% increase from baseline, indicating a greater susceptibility of SHR rats to STZ-induced diabetes. In contrast, the groups that did not receive STZ (G3 and G5) maintained normoglycemic levels, reinforcing that hyperglycemia was exclusively due to the action of streptozotocin [17].

At timepoint t = 2, group G6 (comorbidity group) showed a mean blood glucose of 319.75 ± 64.52 mg/dL, higher than the control group G3, but lower than G4, which may suggest that EG + AC administration interferes with glycemia. Group G7, treated with rosmarinic acid (RA), exhibited a worsening glycemic profile, reaching a mean of 506 ± 58.74 mg/dL, an increase of 31.57% relative to t = 1, although without statistical difference from groups G4 and G6. Group G8, treated with hydrochlorothiazide (HCTZ), showed a mean glucose level of 375.30 ± 64.51 mg/dL, which also did not differ statistically from groups G4 and G6.

Group G5 (SHR with nephrolithiasis but no STZ) remained normoglycemic throughout the experiment, confirming that lithogenesis induced by EG + AC does not directly interfere with carbohydrate metabolism in the absence of STZ.

### 2.3. Urine Results

#### 2.3.1. Calcium Oxalate Crystals in Urine

**Figure 2:**
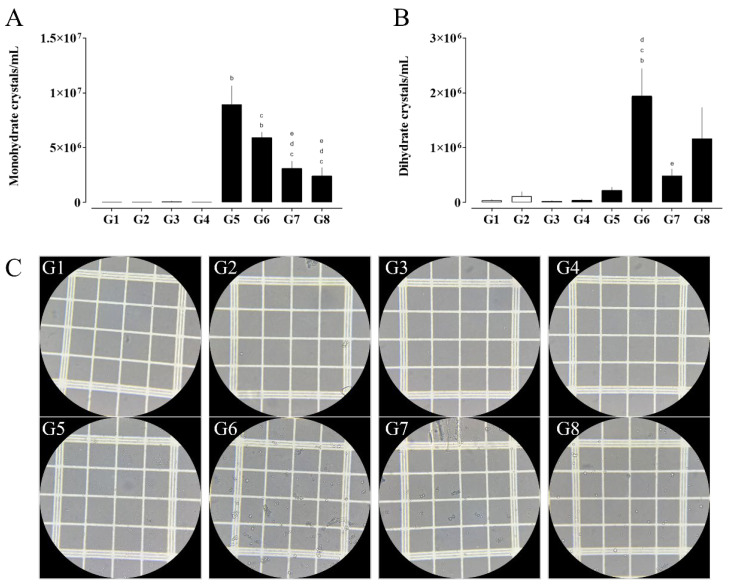
Quantification of calcium oxalate monohydrate (**A**) and dihydrate (**B**) crystals, alongside representative photomicrographs of urinary crystals (**C**, G1–G8). Images were acquired using light microscopy under 400× magnification. (a) G1 vs. G2 and G3; (b) G3 vs. G4, G5, and G6; (c) G4 vs. G6, G7, and G8; (d) G5 vs. G6, G7, and G8; and (e) G6 vs. G7 and G8. Data are expressed as mean ± SEM. Statistical significance was determined by one-way ANOVA followed by Dunnett’s post hoc test. *p* < 0.05 was considered significant.

The analysis of urinary crystal presence revealed important differences among the experimental groups. Regarding monohydrate calcium oxalate crystals (Figure 2A), a significant increase was observed in groups G5 (SHR, EG + AC) and G6 (SHR, STZ, EG + AC) compared to G3 (naive, SHR), indicating that nephrolithiasis induction with ethylene glycol and ammonium chloride (EG + AC) promotes a marked formation of these crystals even in the absence of diabetes. On the other hand, the treated groups, G7 (RA 10 mg/kg) and G8 (HCTZ 5 mg/kg), showed a significant reduction in monohydrate calcium oxalate crystals formation compared to G5 and G6, suggesting a protective effect of these treatments on lithogenesis, even in a context of multiple comorbidities.

Regarding dihydrate calcium oxalate crystals (Figure 2B), a pronounced increase was observed in group G6 compared to groups G3, G4, and G5, whereas group G7 exhibited a significant reduction relative to G6. These results suggest that the combination of hypertension, diabetes, and nephrolithiasis may enhance the formation of dihydrate crystals. Notably, treatment with RA appears to attenuate this increase, indicating a potential protective effect against the formation of this crystal type.

#### 2.3.2. Renal Function Markers in Urine

The evaluation of urinary markers revealed changes consistent with renal dysfunction across different experimental groups [23,24]. Urinary creatinine excretion (Table 1) was significantly reduced in group G6 (SHR, STZ, EG + AC) compared to G3 (naive SHR), and also in groups G6, G7 (RA), and G8 (HCTZ) relative to G5 (SHR, EG + AC). This reduction suggests that the combination of hypertension, diabetes, and nephrolithiasis impairs renal function by decreasing creatinine excretion capacity. Moreover, treatment with RA or HCTZ did not restore this function, indicating that these compounds were not effective in improving glomerular filtration in this experimental model.

Urinary urea excretion was also elevated in groups G2, G4, G6, G7, and G8. The increase in G2 suggests a direct effect of diabetes on nitrogen metabolism. The sustained elevation observed in groups G7 and G8 indicates that treatment with RA and HCTZ did not reduce urea excretion and may have even potentiated it.

Proteinuria also showed relevant alterations. Group G3 exhibited higher levels of urinary proteins compared to G1, reiterating that hypertension alone compromises the glomerular barrier [23,24]. However, groups G6, G7, and G8 showed lower proteinuria compared to G5, which may indicate a different effect of the combined comorbidities (hypertension, diabetes, and nephrolithiasis) and/or protection when associated with treatment. Thus, despite the reduced creatinine excretion, the lower proteinuria observed in these groups suggests a possible antiproteinuric effect of RA and HCTZ, possibly through the reduction in inflammation and/or intraglomerular pressure [25,26].

Glucosuria was consistently elevated in all groups that received streptozotocin (STZ), which was expected after the development of diabetes [27]. A significant increase was observed in group G2 (Wistar + STZ) compared to G1, as well as in groups G4 (SHR + STZ), G6 (SHR + STZ + EG + AC), G7, and G8 relative to their respective controls. Importantly, treatment with RA and HCTZ (groups G7 and G8) had no effect on glucosuria.

#### 2.3.3. Electrolytes and Mineral Metabolism in Urine

Urinary sodium excretion was reduced in group G3 compared to G1, indicating that hypertension may decrease natriuresis as a consequence of sodium retention [28]. This pattern was further exacerbated in group G6 compared to G4, and also observed in group G7 and G8, suggesting that the combination of comorbidities, as well as treatment with HCTZ or RA, contributes to decreased sodium elimination.

Regarding urinary potassium, a significant reduction was observed in groups G5 and G6 compared to G3, which may be attributed to nephrolithiasis and the presence of multiple comorbidities. This reduction was also evident in groups G6 and G8 compared to G4, while groups G7 and G8 showed increased potassium excretion relative to G5. Interestingly, group G7 exhibited greater potassium excretion than G6, suggesting that RA may modulate potassium handling differently compared to HCTZ.

Urinary chloride was significantly reduced in group G3 compared to G1; however, group G6 showed an increase relative to G3, indicating that the combination of hypertension, diabetes, and nephrolithiasis may enhance chloride excretion, possibly due to altered tubular reabsorption or a compensatory diuretic effect induced by the treatments.

Total urinary calcium excretion, normalized by creatinine, was significantly higher in group G2 compared to G1, indicating that STZ-induced diabetes promotes alterations in calcium metabolism. A similar pattern was observed in group G4 relative to G3. Furthermore, groups G6, G7, and G8 exhibited higher levels of urinary calcium than G5, suggesting that the combination of nephrolithiasis and diabetes, along with treatments with RA and HCTZ, increases the excretion of free calcium. This effect was not observed in group G5, where calcium is likely predominantly present in a crystallized form as calcium oxalate.

### 2.4. Serum Results

The analysis of serum biochemical parameters (Table 2) revealed significant alterations related to renal function, hepatic injury, inflammation, lipid profile, and electrolyte homeostasis across the different experimental groups.

#### 2.4.1. Renal Function Markers and Electrolytes

Serum creatinine levels were significantly elevated in the nephrolithiasis group (G5) compared to the hypertensive control (G3), suggesting renal impairment due to crystal formation. Serum urea levels were markedly increased in groups G2, G5, G6, and G8, indicating that diabetes, hypertension, and nephrolithiasis, either alone or combined, induce renal dysfunction. Notably, the elevated urea in G8, despite intra-group variability, suggests a potential aggravating effect of HCTZ on renal function under these conditions.

Uric acid was significantly elevated in G5 compared to both the hypertensive control (G3) and the treated groups (G6, G7, G8). These findings support that nephrolithiasis may reduce uric acid clearance or increase its production, while comorbid groups may exhibit enhanced uricosuria, possibly due to diabetes-associated polyuria.

Serum albumin levels were reduced in diabetic groups (G2 and G4), with a more pronounced decrease in G4 (SHR + STZ), although this reduction does not appear to result from urinary protein loss, since proteinuria was not elevated in these groups. Neither RA (G7) nor HCTZ (G8) treatments were able to prevent albumin loss in this model.

Regarding electrolyte balance, sodium and chloride concentrations differences were not clinically relevant. Potassium levels were elevated in G5, but decreased in G6 relative to G5, suggesting that combined comorbidities in G6 may interfere with potassium homeostasis. An increase in ionized calcium was observed in diabetic groups (G2 and G4) compared to their respective controls, whereas a significant decrease occurred in G6 relative to G4, suggesting that nephrolithiasis negatively impacts calcium homeostasis in the diabetic–hypertensive setting. Both RA (G7) and HCTZ (G8) increased calcium levels compared to G6, indicating potential regulatory effects on calcium metabolism and possible protection against calcium oxalate crystal formation.

#### 2.4.2. Lipid Profile

Total cholesterol levels were increased in G2 compared to G1, as expected in STZ-induced diabetic models. Triglyceride concentrations were also elevated in G2 and G4 relative to their respective controls, reinforcing the presence of diabetes-associated dyslipidemia and its exacerbation by hypertension. Interestingly, the combination of comorbidities in G6 appeared to reduce triglyceride levels significantly compared to G4, suggesting a potential interaction independent of rosmarinic acid treatment.

In contrast, the group treated with hydrochlorothiazide (G8) showed increased total cholesterol and triglycerides relative to G5, as well as elevated HDL levels compared to G5 and G6. This pattern may indicate a trend toward worsening of the lipid profile with HCTZ administration, a finding consistent with previously reported metabolic side effects of thiazide diuretics.

#### 2.4.3. Hepatic Markers

Diabetes and hypertension, whether alone or combined, induced significant hepatic injury. AST levels increased in G2 compared to G1, and in G4 compared to G3, indicating that both diabetes and its combination with hypertension exacerbate liver damage. Notably, the combination of diabetes and hypertension (G4) resulted in the highest elevations of AST, ALT, and alkaline phosphatase, consistent with severe hepatocellular injury and possible cholestasis.

Nephrolithiasis alone (G5) did not markedly affect hepatic function based on any of the evaluated markers. However, when combined with diabetes and hypertension (G6), alkaline phosphatase levels remained elevated, while AST and ALT levels were comparatively reduced. This pattern may indicate a shift from hepatocellular injury to a more cholestatic or subclinical inflammatory profile in the presence of multiple comorbidities.

#### 2.4.4. Inflammatory and Cardiac Markers

The enzyme CK-MB, a marker of cardiac injury, was markedly reduced in the SHR group (G3) compared to the Wistar control group (G1), which may reflect baseline differences due to genetic background or cardiac adaptation in SHR animals. In contrast, group G7 (treated with rosmarinic acid) showed a significant increase in CK-MB levels compared to G5 and G6, suggesting a potential pro-inflammatory or cardiotoxic effect of rosmarinic acid in this model.

LDH levels were significantly increased in groups G2 and G4 compared to their respective non-diabetic controls (G1 and G3), indicating generalized cellular damage induced by diabetes, further aggravated by hypertension. No significant differences were observed among groups for high-sensitivity C-reactive protein (hs-CRP).

#### 2.4.5. Metabolic Stress Indicators

Blood glucose levels were consistently elevated in the STZ-treated groups (G2, G4, G6, G7, G8), confirming the effectiveness of the diabetes induction model. Treatments with RA and HCTZ did not significantly alter glycemia, which is consistent with their known mechanisms of action.

LDH, a nonspecific marker of cellular injury, was elevated in groups G4 and G5 (SHR + diabetes and SHR + nephrolithiasis), but decreased in G6 and G7, suggesting that the combined comorbidities and the effect of RA did not differ significantly in terms of overall tissue damage. In contrast, group G8 (treated with HCTZ) showed an increase in LDH levels, which may indicate that HCTZ either alone or in combination with nephrolithiasis, hypertension, and diabetes exacerbated cellular injury.

### 2.5. Renal Oxidative Stress and Inflammatory Markers

Oxidative stress biomarkers showed a pattern consistent with oxidative damage in control and comorbidity groups, and antioxidant activity in the treated groups (Table 3). Lipid peroxidation (LPO) was elevated in groups G1–G4 and significantly reduced in groups G6–G8, with the lowest level observed in G8. This reduction may be due to severe chronic hyperglycemia, which can decrease mitochondrial metabolic rate in some tissues, thus reducing ROS generation, as well as depletion of lipid substrates (due to weight loss), which limits the lipid content available for peroxidation. Reduced glutathione (GSH) showed no significant differences between groups. Superoxide dismutase (SOD) activity was decreased in G6, reflecting enzymatic exhaustion in response to the combined stress of hypertension, diabetes, and nephrolithiasis, but was restored in the group treated with rosmarinic acid (G7). Glutathione S-transferase (GST) activity was stable in most groups but showed a reduction in G7 and G8.

N-acetyl-β-D-glucosaminidase (NAG) increased in the treated groups, which may reflect late-stage recovery or a specific action of the treatments on the proximal tubules. Nitrite levels were significantly reduced in G5 and G6, suggesting impairment in the nitric oxide (NO) pathway, likely due to decreased eNOS activity or increased NO degradation. Nephrolithiasis may also be associated with local inflammation and renal endothelial dysfunction, resulting in reduced NO release. G8 (HCTZ) had the lowest nitrite concentration, indicating that HCTZ does not protect the NO pathway and may even worsen it—possibly due to volume depletion and compensatory sympathetic activation, or via direct effects on renal vascular function. In contrast, RA (G7) maintained intermediate nitrite levels (23.04 µM), with no significant difference from control groups, suggesting a possible protective effect on the NO pathway.

### 2.6. Histological Analysis

**Figure 3:**
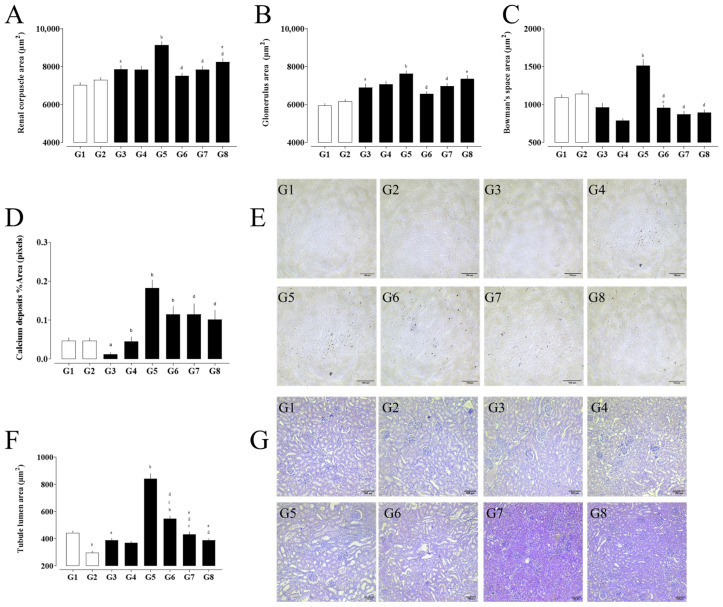
Morphometric analysis of renal structures in experimental groups. (**A**) Renal corpuscle area, (**B**) glomerular tuft area, and (**C**) Bowman’s space area. (**D**) Percentage area of calcium deposits and (**E**) representative Von Kossa-stained kidney sections. (**F**) Tubular lumen area and corresponding (**G**) hematoxylin and eosin (H&E)-stained histological images. (a) G1 vs. G2 and G3; (b) G3 vs. G4, G5, and G6; (c) G4 vs. G6, G7, and G8; (d) G5 vs. G6, G7, and G8; and (e) G6 vs. G7 and G8. Data are expressed as mean ± SEM. Statistical significance was determined by one-way ANOVA followed by Dunnett’s post hoc test. *p* < 0.05 was considered significant.

The morphometric analysis of renal tissue revealed significant structural alterations among the experimental groups (Figure 3). Calcium deposition, which was absent or minimal in the control groups (G1–G4), was markedly increased in the nephrolithiasis-induced groups (G5–G8), with the greatest accumulation observed in G5. Group G5 also exhibited pronounced enlargement of the renal corpuscle, glomerular tuft, and Bowman’s space areas, indicating glomerular hypertrophy and possible capsular distension associated with tissue damage. Treatment with rosmarinic acid (G7) and hydrochlorothiazide (G8) partially attenuated these changes, reducing Bowman’s space and partially normalizing glomerular dimensions.

The distal tubular lumen area was significantly enlarged in G5 and G6, suggesting tubular dilation likely due to obstruction or overload. This parameter returned to baseline levels in the treated groups, particularly G7, indicating a protective effect of the interventions on tubular integrity.

## 3. Discussion

The present study explored, for the first time, the combined effects of hypertension, diabetes mellitus, and nephrolithiasis in a single animal model, providing a pathophysiological framework that closely mimics the clinical complexity frequently observed in patients with metabolic and cardiovascular comorbidities. Previous models have described the coexistence of hypertension and diabetes [17,20], but the addition of experimentally induced nephrolithiasis allowed a more integrated evaluation of renal, metabolic, and hemodynamic dysfunctions. This triple comorbidity model reproduced the synergistic deterioration of renal function and oxidative status commonly seen in high-risk populations.

Hemodynamic analysis confirmed that SHR animals presented persistent hypertension, which was aggravated by the induction of diabetes and nephrolithiasis. Treatment with HCTZ significantly reduced systolic and mean arterial pressures, consistent with its established diuretic, natriuretic and antihypertensive mechanisms [10]. In contrast, RA prevented further increases in blood pressure rather than inducing a marked reduction, suggesting a stabilizing effect. This pattern aligns with reports that RA improves endothelial function and vascular tone primarily through its antioxidant and nitric oxide (NO)-modulating properties [25,26]. The restoration of SOD activity in the RA-treated group supports a role for oxidative stress modulation in its hemodynamic effects.

Fasting glucose remained elevated in all streptozotocin-treated groups, confirming the persistence of the diabetic state. Neither RA nor HCTZ significantly altered glycemia. However, RA may indirectly mitigate hyperglycemia-related tissue injury by attenuating oxidative stress and improving microvascular function, as previously suggested in other diabetic and hypertensive models [11,26]. Indirect metabolic regulatory pathways were not explored in this study. So, future studies should investigate the effects of RA on insulin sensitivity, glucose transport, and hepatic glucose metabolism to better elucidate its potential mechanisms in glycemic regulation.

Renal function parameters revealed that the coexistence of hypertension, diabetes, and nephrolithiasis markedly impaired glomerular filtration, as evidenced by reduced creatinine excretion and increased urea and proteinuria. Notably, both treatments reduced urinary calcium oxalate crystal formation, indicating a protective effect against lithogenesis. This finding is consistent with the reported antilithic and diuretic effects of RA, which may involve stabilization of urinary calcium and modulation of oxidative stress in renal tissue [14,15]. The histological attenuation of tubular dilatation and glomerular hypertrophy in the treated groups reinforces the nephroprotective potential of both RA and HCTZ under metabolic stress.

Interestingly, the reduction in lipid peroxidation observed in the diseased groups may not solely indicate enhanced antioxidant defense. A metabolic impairment and reduced mitochondrial activity can decrease the generation of reactive oxygen species, leading to apparently lower lipid peroxidation levels. In this context, the attenuation of oxidative markers in our comorbid model could partly reflect metabolic suppression rather than true redox balance restoration. Nevertheless, the partial recovery of enzymatic antioxidants (e.g., SOD activity) in RA-treated animals supports a contribution of genuine antioxidant and endothelial protective effects [29,30,31,32].

Serum biochemical and hepatic markers demonstrated that the comorbidity model also induced hepatic dysfunction, as indicated by increased AST and ALT levels, particularly in diabetic–hypertensive animals. RA partially prevented these alterations, supporting its previously described hepatoprotective properties [11,26]. Meanwhile, HCTZ treatment aggravated certain metabolic parameters, such as lipid profile and urea, consistent with its known metabolic side effects [10]. In addition, CK-MB values were markedly reduced in the SHR group (G3) compared to the Wistar control group (G1), which may reflect baseline differences due to genetic background or cardiac adaptation in SHR. In contrast, group G7 (treated with RA) showed a significant increase in CK-MB levels compared to G5 and G6, which may reflect two possible scenarios: a mild cardiac stress secondary to the comorbidity model or, alternatively, a recovery toward normal physiological values, as the levels recorded were closer to those of normotensive Wistar rats. Nonetheless, this unexpected finding warrants further investigation to clarify the underlying mechanisms, as isolated biomarker measurements without accompanying histological or functional cardiac assessments limit the ability to draw definitive conclusions.

In addition to its well-documented antioxidant properties, several studies have shown that RA modulates key molecular targets involved in inflammation and endothelial dysfunction, including NF-κB, Nrf2, and eNOS signaling pathways. Clinically, RA and related phenolic compounds have been associated with improvements in vascular reactivity, glycemic control, renal protection in cardiometabolic disorders, and other relevant benefits in different diseases (for review, see references [25,26]).

Taken together, these results indicate that rosmarinic acid exerts a multifaceted but moderate therapeutic effect in this complex model of cardiometabolic and renal disease. Its benefits are primarily related to the attenuation of oxidative stress, reduction of calcium oxalate deposition, and partial stabilization of renal morphology and vascular function. Although it did not reverse hyperglycemia or fully restore renal biochemical parameters, RA demonstrated relevant renoprotective and antilithogenic activity. These findings highlight the potential of RA as a complementary or adjuvant therapeutic candidate in multifactorial chronic diseases involving hypertension, diabetes, and nephrolithiasis, warranting further mechanistic and translational investigation. A limitation of this study is that toxicity was assessed exclusively through some biomarkers, without complementary evaluations such as organ-specific functional assessments. This restricts a more comprehensive interpretation of potential systemic adverse effects and highlights the need for broader toxicity profiling in future studies.

## 4. Materials and Methods

### 4.1. Comorbidity Model

A comorbidity model was developed in spontaneously hypertensive rats (SHR) by simultaneously inducing diabetes and nephrolithiasis. This model was used to evaluate physiopathological parameters differences between experimental groups, as well as the effects of treatment with rosmarinic acid [15] and hydrochlorothiazide [10].

#### 4.1.1. Animals and Experimental Groups

Male Wistar and spontaneously hypertensive rats (SHR), weighting between 250–350 g, were obtained by the UNIVALI Bioterium (Itajaí, Brazil), and all methodologies and procedures proposed herein were submitted to the Animal Ethics Committee of UNIVALI (CEUA UNIVALI nº 029/24; Approval Date—4 September 2024). Animals were maintained at a controlled ambient temperature (22 ± 2 °C) under a 12-h light/dark cycle, with ad libitum access to food and water until the protocol.

Animals were monitored daily for general condition, food and water intake, grooming behavior, and signs of distress. Predefined humane endpoints included loss of >20% of body weight, persistent hypothermia, severe lethargy, inability to access food or water, or any condition indicative of severe suffering. Animals reaching endpoints were euthanized with an overdose of sodium pentobarbital (150 mg/kg, intraperitoneal), followed by confirmation of death through cessation of cardiac and respiratory activity.

The animals were allocated into eight experimental groups, with eight animals in each group. Allocation was performed by an investigator who was not involved in the experimental procedures, ensuring allocation concealment, as follows:

- G1—Wistar Control (Naïve): Non-treated normotensive Wistar rats.
- G2—Wistar + Diabetes mellitus (DM): Wistar rats with diabetes induced by a single intraperitoneal injection of streptozotocin (STZ).
- G3—SHR Control (Naïve): Untreated spontaneously hypertensive rats (SHR).
- G4—SHR + DM: SHR submitted to STZ-induced diabetes.
- G5—SHR + Nephrolithiasis: SHR with nephrolithiasis induced by administration of ethylene glycol and ammonium chloride.
- G6—SHR + DM + Nephrolithiasis: SHR presenting comorbid DM and nephrolithiasis.
- G7—SHR + Comorbidities + Rosmarinic Acid (RA) 10 mg/kg of body weight (b.w.) treatment: SHR with combined DM and nephrolithiasis, treated with rosmarinic acid.
- G8—SHR + Comorbidities + Hydrochlorothiazide (HCTZ) 5 mg/kg b.w. treatment: SHR with combined DM and nephrolithiasis, treated with hydrochlorothiazide.

#### 4.1.2. Diabetes Mellitus Induction with STZ

Streptozotocin (STZ), dissolved in sodium citrate buffer, pH 4.5, was administered as a single intraperitoneal (i.p.) injection at a dose of 70 mg/kg b.w. per animal in day 0 (t = 1). Animals were considered diabetic when capillary blood glucose levels, measured after an 8-h fasting period, exceeded 250 mg/dL after a 7-day period (t = 2) [17].

#### 4.1.3. Nephrolithiasis Induction with EG + AC

After the confirmation of DM (t = 2), the nephrolithiasis induction protocol was initiated. This involved the administration of 1% ethylene glycol (EG) and 1% ammonium chloride (AC) in the drinking water [18,19,33]. The EG + AC solution was provided for 7 consecutive days. During this period, treatment with rosmarinic acid (RA) and hydrochlorothiazide (HCTZ) were administered once daily by oral gavage at 8:00 a.m. On the final day of EG + AC administration (t = 3), urine samples were collected for analysis.

Rosmarinic acid (RA; ≥96% purity, Sigma-Aldrich, St. Louis, MO, USA) and hydrochlorothiazide (HCTZ; ≥99% purity, Sigma-Aldrich St. Louis, MO, USA) were prepared fresh daily immediately before administration. Both compounds were suspended in a 0.5% tween 80 aqueous vehicle to ensure adequate solubility and homogeneity. Vehicle-treated groups received only a 0.5% tween 80 aqueous solution (10 mL/Kg). The dose of 10 mg/kg for RA was selected based on previous studies [13,14,15].

#### 4.1.4. Arterial Pressure Measure

Systolic blood pressure (SBP), diastolic blood pressure (DBP), and mean arterial pressure (MAP) were measured using tail-cuff plethysmography (Bonther, Ribeirão Preto, SP, Brazil, version 2.7.5). All animals were acclimated to the equipment and the acrylic restraint chamber prior to the first measurement. Blood pressure measurements were obtained at three timepoints: T = 1 (baseline), t = 2 (after the establishment of DM), and t = 3 (following EG + AC administration and treatment).

#### 4.1.5. Glucose Measure

Capillary blood glucose levels were measured using a glucometer (G-Tech, Accumed-Glicomed, Duque de Caxias, RJ, Brazil) with blood obtained from the tail vein. Measurements were performed at the previously described timepoints: T = 1 (baseline), t = 2 (after diabetes mellitus induction), and t = 3 (after EG + AC administration and treatment).

#### 4.1.6. Urine Sample Collection and Analysis

At the final timepoint of the experiment (t = 3), animals were individually placed in metabolic cages equipped with grates to separate urine from feces. They were maintained in the cages for 8 h with free access to water, but without food. Urine volume was measured at the 1st, 2nd, 4th, and 8th hours of collection [14,15,34].

Urinary biochemical parameters, including total calcium/creatinine ratio, and spot sample analyses of urinary chloride, creatinine, glucose, potassium, sodium, proteinuria, and total urea/creatinine ratio, were measured. Measurements of Na^+^, K^+^ and Cl^−^ were performed using the ion-selective electrode (ISE) method. Urea, Creatinine, total calcium, albumin and total protein were measured using enzymatic and colorimetric methods

Calcium oxalate crystals, both monohydrate and dihydrate forms, were quantified using a Neubauer counting chamber under light microscopy.

#### 4.1.7. Tissue Samples and Analysis

Lastly, the animals were anesthetized with ketamine (80 mg/kg) and xylazine (10 mg/kg), administered via i.p. Blood was collected via cardiac puncture, and the heart, liver, and kidneys were excised and weighed. The left kidney was sectioned coronally: one half was fixed for histological analysis, and the other was stored in homogenization buffer for subsequent oxidative stress assays.

Blood samples were divided into three different tubes. One tube containing clot activator and gel separator was used to analyze Na^+^, K^+^, Cl^−^, albumin-corrected total calcium surrogate, urea, creatinine, uric acid, AST, ALT, alkaline phosphatase, total cholesterol, HDL, triglycerides, albumin, CK-MB, and lactate. A second tube containing EDTA was used for complete blood count analysis. The third tube, containing fluoride, was used for lactate dehydrogenase (LDH) determination.

Analyses from the gel separator tubes were performed using the AU480 Chemistry Analyzer (Beckman Coulter, Brea, CA, USA, serial number 2017081340). Measurements of Na^+^, K^+^, Cl^−^, and lactate were performed using the ion-selective electrode (ISE) method. Urea, AST, ALT, alkaline phosphatase, and CK-MB were measured using enzymatic methods. Creatinine, uric acid, total cholesterol, HDL, triglycerides, and albumin were measured using colorimetric methods. Albumin-corrected total calcium, total protein, and total calcium, with the latter two determined by colorimetric methods. The sample containing fluoride for LDH analysis was analyzed using the Stat Profile Prime Blood Gas Analyzer (Nova Biomedical, Waltham, MA, USA, serial number P073210340L).

Organs were homogenized in potassium phosphate buffer (200 mM with pH 6.5; 1:3 *w*/*v*) by maceration and proceeded as described by [35]. Reduced glutathione (GSH) [36], lipid hydroperoxides (LPO) [37,38], superoxide dismutase (SOD) [39,40], glutathione-S-transferase (GST) [41], myeloperoxidase (MPO) [42], N-acetylglucosamine (NAG) [43] and nitrite [44] content was measured following previously mentioned methods [35].

The fixed left kidney was processed for histological staining with hematoxylin and eosin (H&E) and von Kossa techniques to evaluate tissue morphology and calcium deposition, respectively. Five images of each kidney were captured at 10× magnification for each stain. In von Kossa-stained sections, the area percentage of calcium deposits was quantified using ImageJ software (version 1.54p) [45]. In H&E-stained sections, the areas of the renal corpuscle and Bowman’s space were measured. Tubular lumen dilatation was also evaluated by measuring the distal tubule lumen area, using the same software.

### 4.2. Statistical Analysis

All results are expressed as mean ± standard error of the mean (SEM). Statistical analyses were performed by comparing the groups: G1 with G2 and G3 (a); G3 with G4, G5, and G6 (b); G4 with G6, G7, and G8 (c); G5 with G6, G7, and G8 (d); and, G6 with G7 and G8 (e) using one-way or two-way ANOVA, followed by Dunnett’s post hoc test for multiple comparisons. Analyses were performed in GraphPad Prism software (version 8.0.1 for Windows, GraphPad Software, Boston, MA, USA). Differences were considered statistically significant when *p* < 0.05.

## 5. Conclusions

In conclusion, the results demonstrate that blood pressure in SHR animals was further exacerbated by the induction of diabetes and nephrolithiasis. Treatment with rosmarinic acid (RA) effectively prevented the progression of hypertension under these comorbid conditions, stabilizing blood pressure levels, while hydrochlorothiazide (HCTZ), as expected, promoted a significant antihypertensive effect. Isolated nephrolithiasis induced calcium oxalate crystal accumulation in hypertensive animals, and the coexistence of the three comorbidities intensified the formation of calcium oxalate dihydrate crystals while maintaining monohydrate similar levels to the lithogenic SHR group. Both RA and HCTZ reduced crystal formation, suggesting nephroprotective and potentially antilithic effects. These treatments also improved, but did not fully normalize, renal dysfunction markers, as evidenced by decreased proteinuria, reduced calcium deposits, and attenuation of tubular dilatation. Overall, this study uniquely investigated the integrated pathophysiological interactions among hypertension, diabetes mellitus, and nephrolithiasis in an experimental model, alongside the therapeutic potential of RA and HCTZ. To date, no previous studies have simultaneously explored these three comorbidities, which frequently coexist in clinical settings and synergistically accelerate renal and cardiovascular injury. Thus, the findings presented herein provide valuable insights and a solid experimental basis for future therapeutic strategies targeting complex cardiorenal–metabolic disorders.

## Figures and Tables

**Table 1:** Comparison of urinary renal function markers and electrolytes in the experimental groups.

	G1	G2	G3	G4	G5	G6	G7	G8
**Urinary markers**								
Creatinine (mg/dL)	62.38 ± 4.38	41.83 ± 11.11	39.42 ± 5.75	24.77 ± 5.28	37.10 ± 4.64	20.22 ± 2.05 ^b,d^	19.91 ± 3.38 ^d^	14.23 ± 2.05 ^d^
Total Urea (mg/mg Creatinine)	52.63 ± 2.18	98.82 ± 12.55 ^a^	53.38 ± 2.31	118.80 ± 7.75 ^b^	39.50 ± 3.25	90.27 ± 15.70 ^b,d^	139.70 ± 16.00 ^d^	142.40 ± 12.11 ^d^
Proteinuria (mg/dL)	66.00 ± 5.64	44.33 ± 13.92	116.40 ± 15.46 ^a^	51.00 ± 28.86 ^b^	121.00 ± 14.12	57.88 ± 10.23 ^d^	44.86 ± 7.91 ^d^	30.67 ± 9.32 ^d^
Glucose (mg/dL)	55.29 ± 19.60	1172.00 ± 356.70 ^a^	39.08 ± 10.06	1735.00 ± 340.30 ^b^	38.53 ± 9.48	1039.00 ± 389.70 ^b^	1774.00 ± 294.00 ^d^	1228.00 ± 395.50 ^d^
Sodium (mmol/L)	51.25 ± 7.81	53.56 ± 7.32	23.29 ± 6.11 ^a^	38.67 ± 7.25	11.63 ± 2.27	9.88 ± 0.90 ^c^	13.71 ± 1.58 ^c^	27.83 ± 9.55 ^d,e^
Potassium (mmol/L)	140.30 ± 13.62	133.70 ± 26.22	79.43 ± 12.94	84.50 ± 14.75	18.38 ± 3.42 ^b^	19.88 ± 1.55 ^b,c^	50.00 ± 13.47 ^d,e^	47.67 ± 4.23 ^c,d^
Chloride (mmol/L)	46.75 ± 8.46	58.22 ± 10.48	16.71 ± 2.75 ^a^	26.50 ± 3.86	24.00 ± 4.64	66.00 ± 13.13 ^b^	44.71 ± 9.78	71.17 ± 24.85
Total Calcium (mg/mg Creatinine)	0.21 ± 0.04	1.13 ± 0.37 ^a^	0.08 ± 0.01	0.90 ± 0.19 ^b^	0.03 ± 0.01	0.44 ± 0.16 ^d^	0.46 ± 0.10 ^d^	0.38 ± 0.11 ^d^

(a) G1 vs. G2 and G3; (b) G3 vs. G4, G5, and G6; (c) G4 vs. G6, G7, and G8; (d) G5 vs. G6, G7, and G8; and (e) G6 vs. G7 and G8. Data are expressed as mean ± SEM. Statistical significance was determined by one-way ANOVA followed by Dunnett’s post hoc test. *p* < 0.05 was considered significant.

**Table 2:** Comparison of serum biomarkers across experimental groups, including markers of renal function, electrolytes, lipid profile, hepatic function, inflammatory status, and metabolic stress.

	G1	G2	G3	G4	G5	G6	G7	G8
**Serum renal function markers**								
Creatinine (mg/dL)	0.39 ± 0.01	0.43 ± 0.02	0.43 ± 0.03	0.41 ± 0.01	0.89 ± 0.11 ^b^	1.02 ± 0.32	0.49 ± 0.04	0.63 ± 0.24
Urea (mg/dL)	49.23 ± 0.84	63.75 ± 3.05 ^a^	47.31 ± 2.02	63.95 ± 2.50	147.20 ± 30.20 ^b^	151.00 ± 40.90 ^b^	95.00 ± 10.13	176.20 ± 64.87
Uric acid (mg/dL)	2.06 ± 0.16	1.95 ± 0.11	2.11 ± 0.20	1.81 ± 0.23	3.98 ± 0.67 ^b^	1.25 ± 0.33 ^d^	1.34 ± 0.16 ^d^	1.61 ± 0.28 ^d^
Albumin (g/dL)	3.57 ± 0.02	3.36 ± 0.05 ^a^	3.47 ± 0.03	3.05 ± 0.06 ^b^	3.71 ± 0.02	3.15 ± 0.09 ^b,d^	2.90 ± 0.11 ^d^	3.09 ± 0.21 ^d^
**Electrolytes**								
Sodium (mmol/L)	138.90 ± 0.50	137.10 ± 0.68 ^a^	136.90 ± 0.22 ^a^	135.80 ± 0.39	141.30 ± 1.95	133.50 ± 1.85 ^d^	140.00 ± 0.62 ^e^	140.70 ± 1.76 ^e^
Potassium (mmol/L)	5.35 ± 0.12	5.63 ± 0.47	5.22 ± 0.06	5.71 ± 0.19	6.23 ± 0.36 ^b^	5.25 ± 0.21 ^d^	5.41 ± 0.25	5.75 ± 0.26
Chloride (mmol/L)	101.80 ± 0.63	97.33 ± 1.40 ^a^	100.80 ± 0.49	95.22 ± 1.41 ^b^	102.40 ± 1.50	104.00 ± 1.61 ^c^	98.17 ± 2.01	94.33 ± 4.29 ^e^
Calcium (mmol/L)	1.37 ± 0.01	1.42 ± 0.02 ^a^	1.31 ± 0.01 ^a^	1.41 ± 0.01 ^b^	1.33 ± 0.02	1.31 ± 0.03 ^c^	1.43 ± 0.02 ^d,e^	1.42 ± 0.03 ^e^
**Lipid profile**								
HDL (mg/dL)	25.50 ± 1.05	27.22 ± 0.83	19.00 ± 0.44 ^a^	24.50 ± 1.61	18.38 ± 0.98	23.75 ± 2.28	27.57 ± 1.65 ^d^	33.17 ± 3.70 ^d,e^
Total Cholesterol (mg/dL)	67.75 ± 4.51	95.00 ± 10.76 ^a^	49.86 ± 1.03	58.33 ± 3.15	52.88 ± 2.91	58.25 ± 2.70	55.71 ± 1.82	73.00 ± 8.65 ^d^
Triglycerides (mg/dL)	97.88 ± 12.19	714.30 ± 241.20 ^a^	48.00 ± 5.10	151.50 ± 37.18 ^b^	45.38 ± 7.80	79.50 ± 17.17 ^c^	70.57 ± 7.46 ^c^	118.20 ± 30.64 ^d^
**Hepatic markers**								
AST (U/L)	120.40 ± 8.22	179.40 ± 17.22 ^a^	157.00 ± 12.89	466.20 ± 113.10 ^b^	163.50 ± 19.85	126.70 ± 13.43 ^c^	283.80 ± 38.74 ^d,e^	236.60 ± 56.30 ^c^
ALT (U/L)	42.64 ± 1.88	84.79 ± 11.75 ^a^	75.07 ± 6.42 ^a^	260.80 ± 63.81 ^b^	56.71 ± 5.87	68.96 ± 8.42 ^c^	104.60 ± 13.86 ^c,d^	84.12 ± 16.38 ^c^
Alkaline Phosphatase (U/L)	77.83 ± 2.71	282.00 ± 84.99 ^a^	300.10 ± 9.11 ^a^	736.50 ± 84.00 ^b^	119.90 ± 7.37	473.90 ± 134.30 ^d^	682.60 ± 103.90 ^d^	545.70 ± 139.60
**Inflammation markers**								
CK-MB (U/L)	1243.00 ± 163.80	1449.00 ± 172.00	682.70 ± 58.28 ^a^	924.70 ± 116.70	610.70 ± 96.12	476.00 ± 45.93 ^c^	1042.00 ± 130.30 ^d,e^	482.70 ± 77.97 ^c^
LDH (U/L)	94.38 ± 11.02	384.90 ± 127.30 ^a^	300.10 ± 9.11	736.50 ± 84.00 ^b^	119.90 ± 7.37	473.90 ± 134.30	682.60 ± 103.90 ^d^	545.70 ± 139.60 ^d^
hs-PCR (mg/L)	0.02 ± 0.00	0.03 ± 0.00	0.05 ± 0.02	0.03 ± 0.01	0.03 ± 0.00	0.04 ± 0.00	0.03 ± 0.00	0.05 ± 0.00
**Metabolic stress indicators**								
Glucose (mg/dL)	269.70 ± 17.10	520.00 ± 70.48 ^a^	297.70 ± 10.00	666.90 ± 70.23 ^b^	188.70 ± 22.99	450.70 ± 100.70	652.40 ± 74.69 ^d^	586.30 ± 99.75 ^d^
Latic acid (mg/dL)	2.93 ± 0.27	3.34 ± 0.37	2.83 ± 0.47	5.30 ± 0.51 ^b^	4.88 ± 0.75 ^b^	2.55 ± 0.62 ^c,d^	2.54 ± 0.72 ^c,d^	5.52 ± 0.77 ^e^

(a) G1 vs. G2 and G3; (b) G3 vs. G4, G5, and G6; (c) G4 vs. G6, G7, and G8; (d) G5 vs. G6, G7, and G8; and (e) G6 vs. G7 and G8. Data are expressed as mean ± SEM. Statistical significance was determined by one-way ANOVA followed by Dunnett’s post hoc test. *p* < 0.05 was considered significant.

**Table 3:** Oxidative stress and antioxidant defense markers in renal tissue of experimental groups.

	G1	G2	G3	G4	G5	G6	G7	G8
**LPO (µmol/mg of tissue)**	2.72 ± 0.25	1.99 ± 0.24	2.22 ± 0.24	1.67 ± 0.28	1.19 ± 0.10 ^b^	0.93 ± 0.11 ^b,c^	0.99 ± 0.15 ^c^	0.55 ± 0.09 ^c,d^
**GSH (µg/mL)**	4.54 ± 0.16	4.22 ± 0.20	4.28 ± 0.19	4.00 ± 0.42	5.49 ± 0.40	4.84 ± 0.56	4.17 ± 0.32	5.92 ± 0.84
**SOD (U/mg of protein)**	0.31 ± 0.02	0.28 ± 0.01	0.36 ± 0.01 ^a^	0.33 ± 0.03	0.32 ± 0.03	0.21 ± 0.02 ^b,c^	0.36 ± 0.04 ^e^	0.26 ± 0.03
**GST (mmol/min/mg of protein)**	0.94 ± 0.06	0.80 ± 0.05	0.81 ± 0.08	0.77 ± 0.10	0.91 ± 0.06	0.93 ± 0.06	0.76 ± 0.05 ^e^	0.60 ± 0.10 ^d,e^
**NAG (O.D./mg of protein)**	0.40 ± 0.02	0.37 ± 0.02	0.33 ± 0.02 ^a^	0.30 ± 0.00	0.28 ± 0.01	0.29 ± 0.01	0.35 ± 0.01 ^c,d,e^	0.33 ± 0.01 ^d,e^
**MPO (O.D./mg of protein)**	0.01 ± 0.00	0.01 ± 0.00	0.01 ± 0.00	0.01 ± 0.00	0.01 ± 0.00	0.02 ± 0.01	0.01 ± 0.00	0.02 ± 0.01
**Nitrite (µM)**	25.06 ± 0.81	23.64 ± 1.14	28.59 ± 2.38	26.80 ± 0.77	20.26 ± 1.42 ^b^	20.65 ± 1.61 ^b,c^	23.04 ± 1.59	18.87 ± 1.72 ^c^

(a) G1 vs. G2 and G3; (b) G3 vs. G4, G5, and G6; (c) G4 vs. G6, G7, and G8; (d) G5 vs. G6, G7, and G8; and (e) G6 vs. G7 and G8. Data are expressed as mean ± SEM. Statistical significance was determined by one-way ANOVA followed by Dunnett’s post hoc test. *p* < 0.05 was considered significant.

## Data Availability

The raw data supporting the conclusions of this article will be made available by the authors on request.
